# Long-term effect of simulated five years professional mechanical biofilm removal on the luting gap of ceramic restorations

**DOI:** 10.1186/s12903-024-04066-3

**Published:** 2024-03-02

**Authors:** Miriam Cyris, Philipp Holtmann, Christof E. Dörfer, Louise Holtmann, Matthias Kern, Christian Graetz

**Affiliations:** 1 Clinic of Conservative Dentistry and Periodontology, Christian-Albrechts University at Kiel, Kiel, Germany; 2 Department of Prosthodontics, Propaedeutics and Dental Materials, Christian-Albrechts University at Kiel, Kiel, Germany

**Keywords:** Air polishing, Biofilm removal, Loss of substance, Adhesive technique, Dental prophylaxis, Ceramic restoration

## Abstract

**Background:**

Achieving sufficient professional mechanical biofilm removal (PMPR) can be challenging in supportive periodontal therapy (SPT), particularly in patients with prosthetic restorations. This experimental study aimed to simulate five years of SPT with periodic PMPR near the luting gap of ceramic restorations using a rubber cup with polishing paste (RCP), air polishing with two different low-abrasive powders (LAPA-1: glycine powder, LAPA-2: erythritol powder), and non-professional mechanical cleaning (control group) to measure the extent of volume loss in the luting gap after baseline (∆V = V_baseline_-V_1-5_; in µm^3^).

**Methods:**

Two operators randomly performed PMPR ten times for thirty seconds on one of four sides of 30 crown replicas fixed with glass-ionomer cement (CGIZ: *n* = 15) or adhesive bonding (CAB: *n* = 15). The replicas were separated in a template during PMPR, and afterward, cleaned for five seconds per side with a sonic brush under flowing water. The artificial aging process between two PMPRs simulated a 5-year SPT with two PMPRs per year. Profilometric measurements were performed at baseline and after each second PMPR to obtain the mean change of ∆V. The statistical evaluation of the data was carried out using nonparametric tests with Bonferroni correction applied for multiple tests.

**Results:**

Ninety-six out of 120 sides could be included in the analysis. PMPR methods showed a loss of substance in the luting gap with a ∆V (mean(standard deviation)) of -4.35 × 10^6^(4.8 × 10^6^)µm^3^ versus 8.79 × 10^4^(1.05 × 10^6^)µm^3^ for control at V_5_ (*p* ≤ 0.001). No significant differences of ∆V_1-5_ values could be identified in the control (*p* > 0.05), whereat all PMPRs showed a significant increasing loss of substance per simulated year (*p* ≤ 0.001). Intergroup comparison identified LAPA-1 as having the highest significant loss of substance determined on CAB (∆V: -1.05 × 10^7^ (7,2 × 10^6^) µm^3^), followed by LAPA-2 on CAB (∆V: -6.29 × 10^6^ (4,24 × 10^6^) µm^3^), LAPA-1 on CGIZ (∆V: -4.15 × 10^6^ (3,25 × 10^6^) µm^3^), LAPA-2 on CGIZ (∆V: -3.0 × 10^6^ (2,23 × 10^6^) µm^3^), RCP on CAB (∆V: -1.86 × 10^6^ (2,23 × 10^6^) µm^3^) and CGIZ (∆V: -1.2 × 10^6^ (1,31 × 10^6^) µm^3^; *p* ≤ 0.001)).

**Conclusions:**

Within study limitations, all PMPRs caused a significantly higher loss of substance in the luting gap versus control without professional intervention, with the highest values in the CAB group for LAPA-1, LAPA-2 and RCP. Similar findings were observed for CGIZ, although the loss values were lower.

## Background

Periodontitis is a multifactorial inflammatory disease associated with dysbiotic biofilm and characterized by progressive destruction of the periodontium [1]. An adequate periodontal therapy could restore the biocompatibility of the previously diseased root surfaces [2, 3], allowing the reattachment of adjacent tissues [4–7]. Success occurs only if supportive periodontal therapy (SPT) followed by regular appointments for professional mechanical biofilm removal (PMPR) [8]. Additionally, PMPR should be performed as a preventive professional intervention, for example, during orthodontic therapy [9, 10], as orthodontic appliances hinder thorough oral hygiene, leading to the accumulation of biofilm and alteration of the oral microbiome. Similar could be hypothesized for prosthetic restorations or fillings with luting/ filling gap near the marginal gingiva if the patients are non-compliant with self-performed plaque control [11].

Consequently, (1) several visits for PMPR are necessary in a patient’s lifetime to prevent (further) gingival and periodontal inflammation and (2) should be performed with special attention to teeth with exposed root surfaces or residual pockets [12]. Lately, air polishing (AP) has been preferred to remove nonmineralized biofilm as a fast and reliable method with a high level of comfort for both the patient and the operator [13, 14]. In particular, low-abrasiveness powder air polishing (LAPA; e.g. glycine or erythritol powder) is recognized as a minimally invasive tool for the management of biofilms colonizing tooth and root surfaces [15]. It promises effective PMPR while preserving the integrity of the root surface and soft tissue [16], especially in SPT performing PMPR with LAPA seemed to require a shorter treatment time and exhibit a more favorable patient perception than the conventional approach [17]. However, some studies measured loss of hard structure (dentin, cementum) [18] or of restorative materials (glass-ionomer cement, compomer) [19, 20]. Beside the fact that the extent of loss was oftentimes statistically significant in these experimental studies, from a clinical point of view, the question is: how could this lead to a critical side effect of LAPA when repeated PMPRs are performed over extended periods of time, especially in the luting gap of restorations or orthodontic brackets?

Therefore, the aim of this experimental study was to simulate five years of PMPR near the luting gap of ceramic restoration replicas using a rubber cup with polishing paste (RCP) in a prophy contra angle, LAPA with low-abrasive powder (LAPA-1: glycine powder, LAPA-2: erythritol powder) versus non-professional mechanical cleaning (control group) to measure the extent of cement loss in the luting gap. The study hypothesis is therefore that the PMPR carried out with the investigated low-abrasive powders does not cause a significantly higher substance removal of cement in the luting gap of indirect restorations than the application of polishing cup and paste.

## Methods

Before the study, both operators (C.G. and M.C.) trained and calibrated each other in a clinical setting to perform PMPR under the experimental conditions. Both were employees of the Department of Periodontology, Christian-Albrechts University Kiel and had seven to twenty years of professional experience. They had completed the same training program, which included lectures on the applicable theoretical information according to our clinical guidelines and the manufacturer’s guidelines, as well as clinical practical sessions before the test. Although a training session was completed for air flow, it was not possible to calibrate using a parameter due to the method. Additionally, all operators were calibrated for application pressure (1.5 for RCP according Reinhart, Singh-Husgen [20]) using a scale during theoretical training sessions, but no measurements of root surface destruction or roughness were taken during testing.

The frequency of the instruments and the test replicas, as well as the order of the tested instruments, were randomized (Microsoft Excel 16, Microsoft Corporation, One Microsoft Way Redmond, WA, USA) for each operator to exclude any influences of laterality or training effects. Operators instrumented the replicas for a maximum of thirty seconds with each PMPR method.

### Experimental setup

All operators had the same setup and instruments for PMPR near the luting gap of ceramic restorations: (1) for RCP a rubber cup (proxeo TWIST Prophy Cups, W&H Dentalwerk, Bürmoos, Austria; level of hardness: hard) in a prophy contra angle piece (proxeo TWIST WP-66 W, W&H Dentalwerk, Bürmoos, Austria) with polishing paste (Prophy Paste, ProphyCare, DIRECTA AB, Upplands Väsby, Sweden; relative dentin abrasion value: 170) at a rotational speed of 2500 rpm (rounds per minute), (2) the LAPA-1 air-polishing device (LM-ProPower, LM-Instruments Oy, Parainen, Finland) on the middle level with a supragingival nozzle (LM-Supra A nozzle, universal, LM-Instruments Oy, Parainen, Finland) and (3) for LAPA-2 an air-polishing device (AIRFLOW PROPHYLAXIS MASTER, EMS, Nyon, Switzerland) at level 7 with a supragingival nozzle (AIRFLOW MAX handpiece, EMS, Nyon, Switzerland). For RCP, only new instrument tips were used for each operator and trial. The abrasive material of the paste is pumice with a particle size smaller than 88 µm according manufactory information. LAPA-1 was utilized with glycine powder with a particle size of 18–22 µm (KaVo PROPHYflex Perio Powder, KaVo Dental GmbH, Biberach, Germany) and LAPA-2 with erythritol powder with a particle size of 14 μm (AIR-FLOW PLUS powder, EMS, Nyon, Switzerland. After each PMPR, all control sides were cleaned for 5 s under running water with a sonic brush (GUM Playbrush, SUNSTAR Suisse S.A., Etoy, Switzerland) to remove any residual polishing paste or powder.

**Fig. 1:**
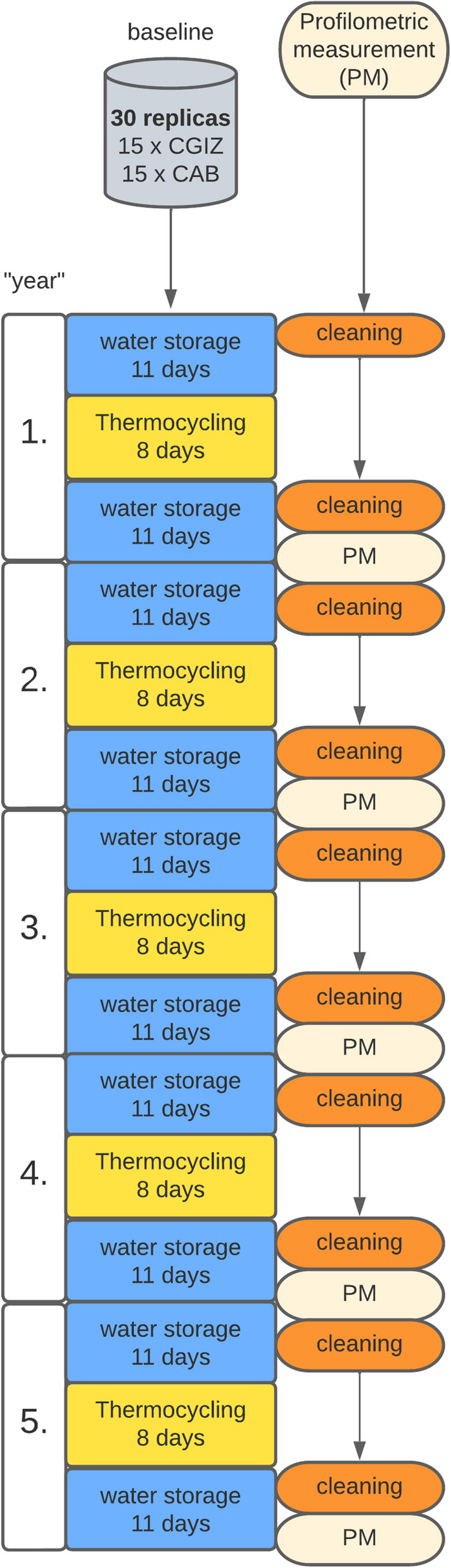
Illustration of the artificial aging and study process

Between each PMPR visit, an artificial aging process was performed (Fig. 1). Thirty crown replicas were subjected to artificial aging during the study, two cement groups with 15 specimens each. This followed a fixed protocol of 150 days. For this purpose, the replicas were subjected to five thermocycling phases (SD Mechatronik, Feldkirchen-Westerham, Germany) of 8 days each. A single phase comprised 7500 cycles, which included an alternation from 5 to 55 °C with a dwell time of 30 s in the respective water bath. In the intervening periods, the replicas were stored in distilled water at 37°C for 22 days each (Thermo Fisher Scientific Precision GP2, Waltham, USA).

### Test teeth

**Fig. 2:**
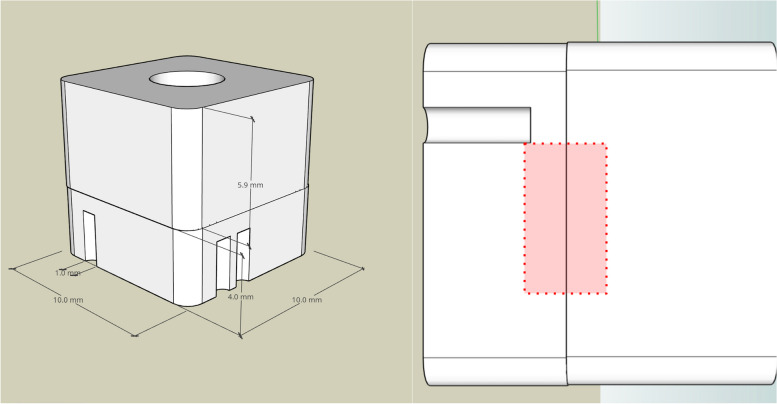
Illustration of the zirconia replicas. Illustration of the dimension of the zirconia replicas (*n* = 30) including a magnification of the area of interest of each side (*n* = 4) at the luting gap

Using the subtractive method, modified test teeth were created from zirconia that replicated the area of the crown luting gap (Fig. 2). The specimens had an average surface roughness Ra of 0.35 ± 0.07 µm.

A simplified model of test teeth was created. For this purpose, a lower part (tooth) and an upper part (crown), which together form a cube, were modeled using 3D software (SketchUp Free, Trimble Inc, Sunnyvale, USA) and represented the replica. In addition, an individual mark in the form of one to four notches was modeled into each of the four sides, which both allowed unique identification of the sides to the respective cleaning method and provided a reference mark for later measurement under the microscope. The distance between the two parts in the area of interest was adjusted to result in a luting gap of 100 µm.

The replicas were milled (Ceramill Matik, Amann-Girrbach, Koblach, Austria) of zirconia ceramic (Zolid Gen-X, Amann-Girrbach, Koblach, Austria) and then sintered according to the manufacturer´s specifications. The replicas were air-abraded with alumina powder (25 µm grain size at 1.5 bar pressure) on the retention surfaces before cementation. Afterward, the objects were cleaned in alcohol in an ultrasonic bath for 30 s and dried with oil-free air. Both parts were cemented under constant pressure (5 kg) with a suitable device according to the instructions of the respective manufacturer. One group (15 × CGIZ) was cemented with glass ionomer cement (Ketac Cem, 3M, Seefeld, Germany), while the other group (15 × CAB) was luted with a composite resin (RelyX Unicem 2, 3M, Seefeld, Germany). Excess cement was removed with a foam pellet for CGIZ and the surfaces of replicas that had been cemented with AD were light-cured for 2 s each before removing excess cement with a scaler according to the manufacturer's instructions. To achieve a uniformly smooth transition between the two parts of the crown replica, the transitions were smoothed with abrasive paper (1200 grit) on a disc grinder (Buehler Automet 250, ITW Test & Measurement, Leinfelden-Echterdingen, Germany) under running water and then polished with diamond polishing paste (MetaDi Supreme 3 µm, ITW Test & Measurement, Leinfelden-Echterdingen, Germany).

### Profilometric measurement

**Fig. 3:**
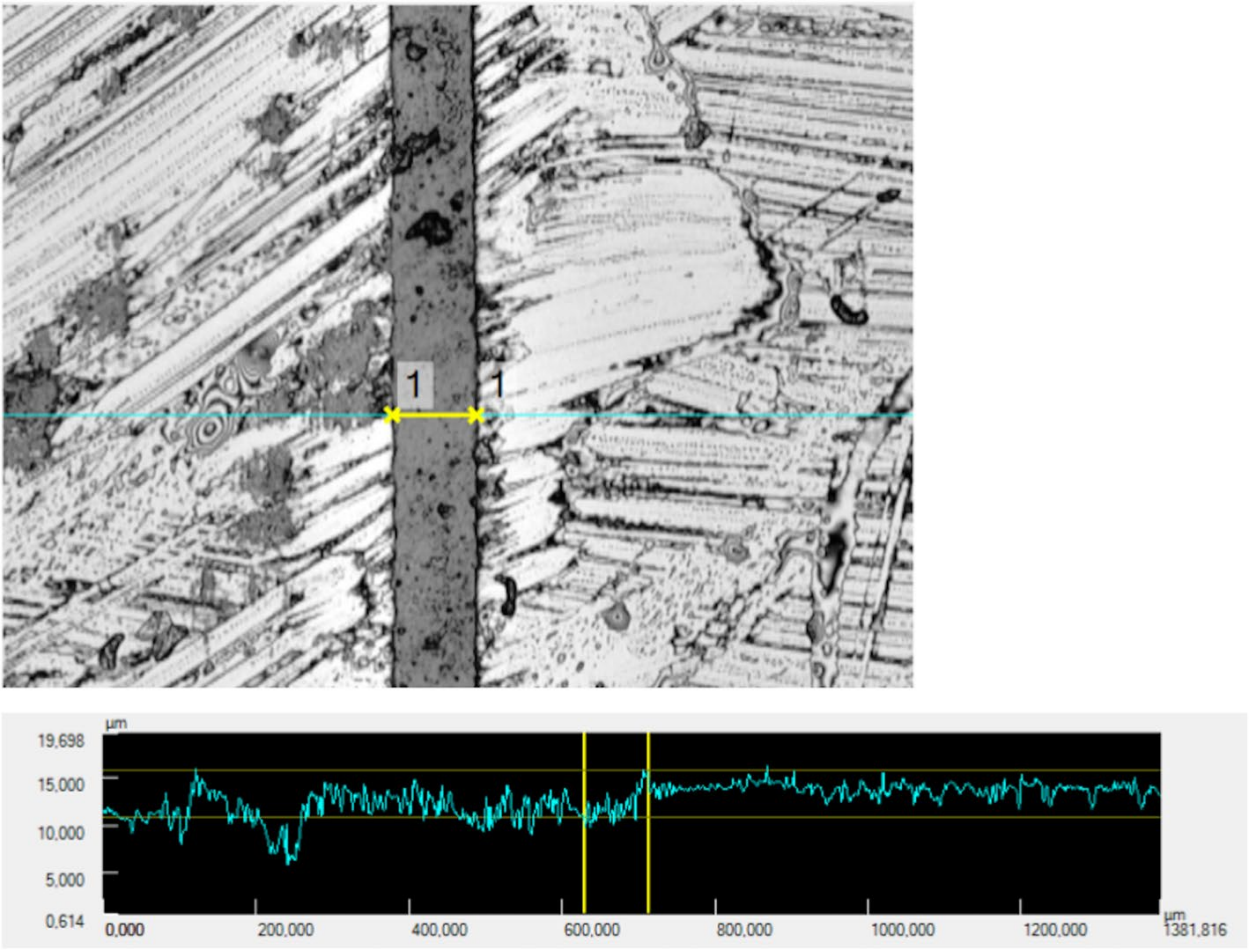
Image of a luting gap width measurement. Example of width measurement of the luting gap: Visually, the luting gap can be clearly distinguished from the ceramic surfaces. In this case, the width of the luting gap (indicated by the yellow arrow) is 83.8 µm. The height difference between the adjacent ceramic surfaces (marked by the two vertical yellow lines) is 4.8 µm

**Fig. 4:**
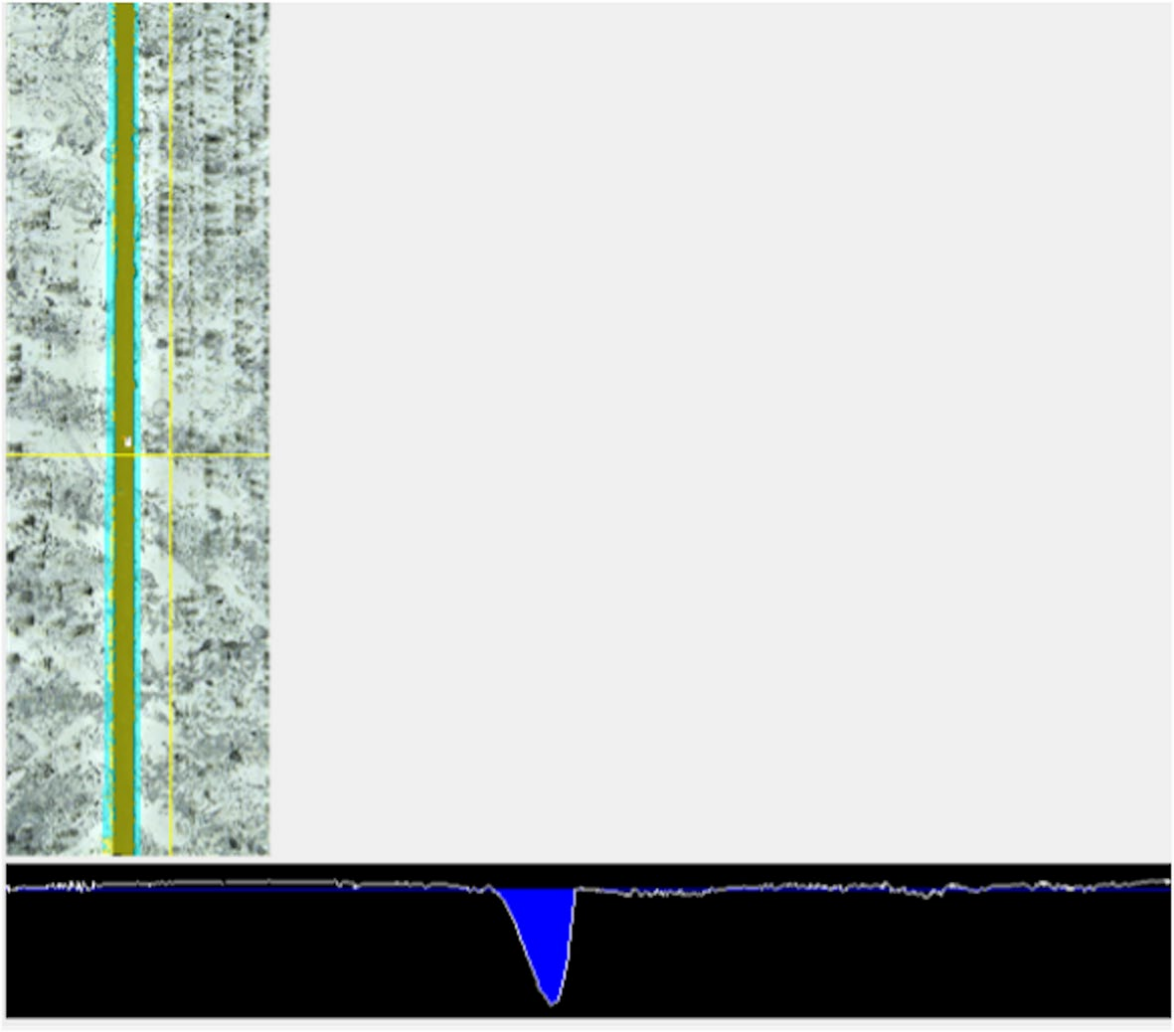
Image of a volume measurement. Example of a volume measurement: The luting gap was marked over its entire length and the height of the ceramic surfaces was set as the threshold value. The blue area inside the graph indicates the acquired volume

The replicas were subjected to a profilometric measurement under the laser scanning microscope (Keyence VK-X-100 series, Keyence Corporation, Osaka, Japan) at 10 × magnification. A measuring field of 4.63 × 1.38 mm was recorded using analytical software (VK Analyse Modul Plus v. 3.8., Keyence Corporation, Osaka, Japan), whereby the upper limit of the measuring range was always formed by the uppermost milled reference mark. The luting gap was measured centrally in the measuring field (Fig. 3). The width of the luting gap was measured at eight reproducible points per side. Measurement of the width of the luting gap was carried out at the beginning (baseline) and after the completion of the test series. To determine the volume change in the luting gap, i.e. a potential removal of cement, the area of the luting gap was marked and the height of the ceramic surfaces was set as a threshold (Fig. 4). Profilometric measurements of the volume were taken at the beginning (baseline) and after each simulated year, six times in total. This was done by one person (P.H.) at two different time points.

### Outcomes and statistical analysis

As a primary outcome, the mean change of the volume of the luting gap was determined and measured as the difference in the area of the crown gap baseline and after each second PMPR (∆V = V_baseline_-V_1-5_; in µm^3^) for each test side (*n* = 120) instrumented by both operators. A secondary outcome was to measure the width of the luting gap at baseline. We planned before the study started, that all sides of luting gap with a mean(standard deviation) of 100(70) µm should be included in the analysis as an ideal luting gap, as mentioned by Hmaidouch, Neumann [21] for different luting methods of ceramic crowns. A narrow luting gap would entail the risk of interference fit, which could lead to increased stress-induced fracture susceptibility for ceramic restorations as used in our study, and on the other side, a wide luting gap could lead to higher washout at the tooth-ceramic junction which may also promote the formation of ceramic fractures and have a detrimental effect on the longevity of the restoration [22].

The results were separated for the three types of instrumentation (RCP, LAPA-1, LAPA-2) and the control sides without PMPR. The investigator (P.H.) was blinded to the instruments and operators when performing the planimetric evaluation. The Spearman-rho was used to test the reability of the profilometric measurements of the volume of the luting gap, as the measurements were performed by the same rater (P.H.) at two different time points.

Before the investigation, we performed a sample size calculation using data from a comparable investigation [23] and found *n* = 96 test sides to be sufficient to detect an RCE difference of less than 5% between the groups of instruments and control (power of 80%). After pretests, we increased the number to *n* = 120 according to, e.g. measuring failures, loss of adhesion or fractures of the replicas during artificial aging.

Aside from a descriptive evaluation of the data, a statistical analysis of the measurements was performed with statistical software (SPSS Statistics 24, IBM, Chicago, IL, USA). The normal distribution was confirmed using the Shapiro–Wilk test. Differences between experience groups were analyzed using Kruskal–Wallis test and for intragroup volume changes the Friedman test. Post hoc tests were performed using the Mann–Whitney U test with a Bonferroni correction to adjust for the effects of multiple testing. For pairwise comparisons, the Wilcoxon signed-rank test was used. All tests were two-sided; statistical significance was assumed if *p* ≤ 0.05.

## Results

In total, 96 sides (CGIZ: *n* = 52; CAB: *n* = 44) out of 120 sides could be analyzed for ∆V (distribution per PMPR group: LAPA-1 *n* = 23, LAPA-2 *n* = 24, RCP *n* = 26 and 23 sides in control [no PMPR]). Therefore, twenty-four sides were excluded from statistical analysis due to unacceptable deviations of the baseline gap width with results lower than 30 µm or wider than 170 µm (*n* = 17), failure of measuring the volume in the gap (*n* = 3) and fracture of one replica outside study intern reasons (*n* = 4). No decementation occurred.

No normal distribution was detected for ∆V (Shapiro–Wilk; *p* < 0.001). According to the reability of our measurement, we found a Spearman-rho of 0.970 (*p* ≤ 0.001), that showed a high correlation of 78 randomized repeated volume measurements.

The width of the luting gap was in mean(standard deviation) 75.90(19.91) µm at baseline and after simulation of five years through artificial aging 80.45(22.10) µm, without significant difference neither among all cleaning groups (*p* > 0.05) nor between both types of cementation (*p* > 0.05), where the width of the luting gap showed a statistically significant increase in LAPA-1 between baseline and end of observation time (CGIZ: *p* = 0.004; CAB: *p* = 0.003), for control group in CGIZ (*p* = 0.039) and for RCP in CAB (*p* = 0.005). For more details, see Tables 1 and 2.

**Table 1:** Overview according to change of the volume (∆V in µm^3^) in the luting gap after simulated 1, 2, 3, 4 and 5 years of artificial aging as well as per each simulated aging year (baseline—1st, 1st—2nd, 2nd – 3rd, 3rd—4th, 4th – 5th) with pairwise instrument comparisons. Also included are the width of the luting gap (GW in µm) at baseline and after 5 years of artificial aging

Cementation type	Instrument	∆V_1_ in µm^3^ (mean ± SD)	∆V_2_ in µm^3^ (mean ± SD)	∆V_3_ in µm^3^ (mean ± SD)	∆V_4_ in µm^3^ (mean ± SD)	∆V_5_ in µm^3^ (mean ± SD)	Pairwise instrument comparison	*p*-value for ∆V_1_	*p*-value for ∆V_2_	*p*-value for ∆V_3_	*p*-value for ∆V_4_	*p*-value for ∆V_5_
**CGIZ**	**LAPA-1** **(*n* = 12)**	-2.57 × 10^5^ (6.75 × 10^5^)	-9.47 × 10^5^ (1.10 × 10^6^)	-1.76 × 10^6^ (2.02 × 10^6^)	-3.12 × 10^6^ (3.20 × 10^6^)	-4.15 × 10^6^ (3.25 × 10^6^)	vs. LAPA-2	1.000	1.000	1.000	1.000	1.000
							vs. RCP	1.000	0.705	0.188	0.024	0.075
							vs. control	1.000	0.127	**0.002**	** < 0.001**	** < .0.001**
	**LAPA-2** **(*n* = 13)**	-4.20 × 10^5^ (7.67 × 10^5^)	-6.04 × 10^5^ (1.12 × 10^6^)	-1.58 × 10^6^ (1.55 × 10^6^)	-2.95 × 10^6^ (2.21 × 10^6^)	-3.00 × 10^6^ (2.23 × 10^6^)	vs. RCP	1.000	1.000	0.390	0.139	0.467
							vs. control	1.000	0.862	**0.007**	**0.002**	** < 0.001**
	**RCP** **(*n* = 14)**	-1.44 × 10^5^ (5.24 × 10^5^)	-3.52 × 10^5^ (1.20 × 10^6^)	-5.00 × 10^5^ (8.18 × 10^5^)	-9.02 × 10^5^ (1.18 × 10^6^)	-1.20 × 10^6^ (1.31 × 10^6^)	vs. control	1.000	1.000	0.840	0.811	0.144
	**control** **(*n* = 13)**	-1.93 × 10^4^ (4.73 × 10^4^)	2.11 × 10^5^ (1.24 × 10^6^)	3.40 × 10^5^ (1.36 × 10^6^)	4.19 × 10^5^ (1.32 × 10^6^)	3.44 × 10^5^ (1.18 × 10^6^)						
**CAB**	**LAPA-1** **(*n* = 12)**	-1.30 × 10^6^ (2.13 × 10^6^)	-3.36 × 10^6^ (4.18 × 10^6^)	-6.49 × 10^6^ (4.75 × 10^6^)	-9.41 × 10^6^ (6.96 × 10^6^)	-1.05 × 10^7^ (7.20 × 10^6^)	vs. LAPA-2	1.000	0.662	1.000	1.000	1.0000
							vs. RCP	1.000	1.000	0.033	0.024	**0.004**
							vs. control	0.425	**0.018**	**0.001**	** < 0.001**	** < 0.001**
	**LAPA-2** **(*n* = 10)**	-2.36 × 10^6^ (2.83 × 10^6^)	-5.24 × 10^6^ (3.15 × 10^6^)	-6.16 × 10^6^ (3.96 × 10^6^)	-6.18 × 10^6^ (4.14 × 10^6^)	-6.29 × 10^6^ (4.24 × 10^6^)	vs. RCP	0.742	**0.038**	**0.030**	0.139	0.147
							vs. control	**0.015**	** < 0.001**	**0.001**	**0.002**	**0.001**
	**RCP** **(*n* = 12)**	-6.03 × 10^5^ (1.03 × 10^6^)	-1.22 × 10^6^ (1.55 × 10^6^)	-1.09 × 10^6^ (1.86 × 10^6^)	-1.80 × 10^6^ (2.09 × 10^6^)	-1.86 × 10^6^ (2.23 × 10^6^)	vs. control	0.626	0.403	1.000	0.811	0.650
	**control** **(*n* = 10)**	4.89 × 10^4^ (3.93 × 10^5^)	-4.13 × 10^4^ (3.98 × 10^5^)	-9.22 × 10^4^ (5.31 × 10^5^)	-1.87 × 10^5^ (6.90 × 10^5^)	-2.46 × 10^5^ (7.72 × 10^5^)						
		**∆V**_**1st**_** in µm**^**3**^** (mean ± SD)**	**∆V**_**2nd**_** in µm**^**3**^** (mean ± SD)**	**∆V**_**3rd**_** in µm**^**3**^** (mean ± SD)**	**∆V**_**4th**_** in µm**^**3**^** (mean ± SD)**	**∆V**_**5th**_** in µm**^**3**^** (mean ± SD)**		***p***-value for ∆V_**1st**_	***p***-value for ∆V_**2nd**_	***p***-value for ∆V_**3rd**_	***p***-value for ∆V_**4th**_	***p***-value for ∆V_**5th**_
**CGIZ**	**LAPA-1** **(*n* = 12)**	-2.57 × 10^5^ (6.75 × 10^5^)	-6.90 × 10^5^ (6.06 × 10^5^)	-8.19 × 10^5^ (1.38 × 10^6^)	-1.35 × 10^6^ (1.55 × 10^6^)	-1.03 × 10^6^ (7.45 × 10^5^)	vs. LAPA-2	1.000	1.000	1.000	1.000	1.000
							vs. RCP	1.000	1.000	1.000	0.763	0.371
							vs. control	1.000	1.000	0.293	**0.018**	**0.002**
	**LAPA-2** **(*n* = 13)**	-4.20 × 10^5^ (7.67 × 10^5^)	-1.84 × 10^4^ (6.02 × 10^5^)	-9.81 × 10^5^ (9.58 × 10^5^)	-1.36 × 10^6^ (1.28 × 10^6^)	-5.11 × 10^4^ (1.17 × 10^6^)	vs. RCP	1.000	1.000	0.768	0.213	0.609
							vs. control	1.000	1.000	0.172	**0.002**	**0.003**
	**RCP** **(*n* = 14)**	-1.44 × 10^5^ (5.24 × 10^5^)	-2.07 × 10^5^ (8.62 × 10^5^)	-1.48 × 10^5^ (8.50 × 10^5^)	-4.01 × 10^5^ (5.89 × 10^5^)	-3.00 × 10^5^ (6.11 × 10^5^)	vs. control	1.000	1.000	1.000	0.750	0.360
	**control** **(*n* = 13)**	-1.93 × 10^4^ (4.73 × 10^5^)	2.30 × 10^5^ (1.04 × 10^6^)	1.29 × 10^5^ (7.41 × 10^5^)	7.83 × 10^4^ (5.12 × 10^5^)	-7.42 × 10^4^ (6.96 × 10^5^)						
**CAB**	**LAPA-1** **(n = 12)**	-1.30 × 10^6^ (2.13 × 10^6^)	-2.05 × 10^6^ (2.65 × 10^6^)	-3.13 × 10^6^ (3.01 × 10^6^)	-2.91 × 10^6^ (4.15 × 10^6^)	-1.12 × 10^6^ (1.86 × 10^6^)	vs. LAPA-2	0.281	0.492	1.000	1.000	1.000
							vs. RCP	1.000	1.000	**0.050**	**0.029**	**0.006**
							vs. control	0.690	0.064	**0.002**	** < 0.001**	** < 0.001**
	**LAPA-2** **(*n* = 10)**	-2.36 × 10^6^ (2.83 × 10^6^)	-2.87 × 10^6^ (2.27 × 10^6^)	-9.17 × 10^5^ (3.11 × 10^6^)	-2.51 × 10^4^ (1.17 × 10^6^)	-1.09 × 10^5^ (1.21 × 10^6^)	vs. RCP	0.210	0.053	**0.020**	0.120	0.139
							vs. control	**0.004**	** < 0.001**	**0.001**	**0.001**	**0.002**
	**RCP** **(*n* = 12)**	-6.03 × 10^5^ (10.3 × 10^6^)	-6.21 × 10^5^ (7.50 × 10^5^)	1.24 × 10^5^ (7.30 × 10^5^)	-7.09 × 10^5^ (9.89 × 10^5^)	-5.97 × 10^4^ (6.83 × 10^5^)	vs. control	0.875	0.566	1.000	0.733	0.729
	**control** **(*n* = 10)**	4.89 × 10^4^ (3.93 × 10^5^)	-9.02 × 10^4^ (1.72 × 10^5^)	-5.08 × 10^4^ (2.52 × 10^5^)	-9.52 × 10^4^ (2.39 × 10^5^)	-5.85 × 10^4^ (2.87 × 10^5^)						
**Cementation type**	**Instrument**	**GW**_**1**_** in µm (mean(SD))**		**GW**_**5**_** in µm (mean(SD))**	**p-value for GW**_**1**_** vs. GW**_**5**_		**Pairwise instrument comparison**	***p***-value for gap_**1**_				***p***-value for gap_**5**_
**CGIZ**	**LAPA-1** **(*n* = 12)**	76.19(18.70)		81.85(21.14)	**0.004**		vs. LAPA-2	1.000				1.000
							vs. RCP	1.000				1.000
							vs. control	1.000				1.000
	**LAPA-2** **(*n* = 13)**	74.00(22.36)		73.44(19.99)	0.753		vs. RCP	1.000				1.000
							vs. control	1.000				1.000
	**RCP** **(*n* = 14)**	75.13(15.12)		78.04(18.85)	0.510		vs. control	1.000				1.000
	**control** **(*n* = 13)**	72.16(24.98)		78.01(23.36)	**0.039**							
**CAB**	**LAPA-1** **(*n* = 12)**	81.22(26.14)		90.04(28.28)	**0.003**		vs. LAPA-2	1.000				1.000
							vs. RCP	1.000				1.000
							vs. control	1.000				1.000
	**LAPA-2** **(*n* = 10)**	77.77(12.91)		76.99(24.58)	0.878		vs. RCP	1.000				1.000
							vs. control	1.000				1.000
	**RCP** **(*n* = 12)**	74.92(18.11)		83.89(21.56)	**0.005**		vs. control	1.000				1.000
	**control** **(*n* = 10)**	76.84(21.32)		82.23(20.72)	0.203							

*CAB* Crown replicas with adhesive bonding, *CGIZ* Crown replicas with glass-ionomer cement, *GW* Gap width, *LAPA-1* Air polishing with glycine powder, *LAPA-2* Air polishing with erythritol powder, *RCP* Rubber cup with polishing paste, standard deviation (SD) and *p*-values of ∆V were calculated over an N of 96 for different crown surfaces

**Table 2:** Overview according to the distribution N of change of the volume (no or decreasing volume vs. increasing volume) in the luting gap after simulated 5 years of artificial aging with pairwise instrument comparisons

Cementation type	Instrument	N of sites with increasing volume after 5 years artificial aging	N of sites with stable or decreasing volume (%) after 5 years artificial aging	Pairwise instrument comparison	*p*-value for ∆V_5_
**CGIZ**	**LAPA-1** **(*n* = 12)**	12 (100%)	0 (0%)	vs. LAPA-2	1.000
				vs. RCP	1.000
				vs. control	** < 0.001**
	**LAPA-2** **(*n* = 13)**	12 (92.3%)	1 (7.7%)	vs. RCP	1.000
				vs. control	**0.001**
	**RCP** **(*n* = 14)**	12 (85.7%)	2 (14.3%)	vs. control	**0.005**
	**control** **(*n* = 13)**	4 (30.8)	9 (69.2%)		
**CAB**	**LAPA-1** **(*n* = 12)**	12 (100%)	0 (0%)	vs. LAPA-2	1.000
				vs. RCP	1.000
				vs. control	**0.010**
	**LAPA-2** **(*n* = 10)**	10 (100%)	0 (0%)	vs. RCP	1.000
				vs. control	**0.015**
	**RCP** **(*n* = 12)**	10 (83.3%)	2 (16.7%)	vs. control	0.212
	**control** **(*n* = 10)**	5 (50%)	5 (50%)		

*CAB* Crown replicas with adhesive bonding, *CGIZ* crown replicas with glass-ionomer cement, *LAPA-1* Air polishing with glycine powder, *LAPA-2* Air polishing with erythritol powder, *RCP* Rubber cup with polishing paste, standard deviation (SD) and *p*-values of ∆V were calculated over an N of 96 for different crown surfaces

Overall, all test methods showed a loss of substance in the luting gap with a ∆V (mean(standard deviation)) of -4.35 × 10^6^(4.8 × 10^6^)µm^3^ versus 8.79 × 10^4^(1.05 × 10^6^)µm^3^ for control at V_5_ (*p* ≤ 0.001). Additionally, no significant differences for all ∆V_1-5_ values in the control group could be identified (*p* > 0.05) versus significant differences for ∆V_1-5_ values of all PMPRs (*p* ≤ 0.001) with an increasing trend of substance loss per simulated year.

**Fig. 5:**
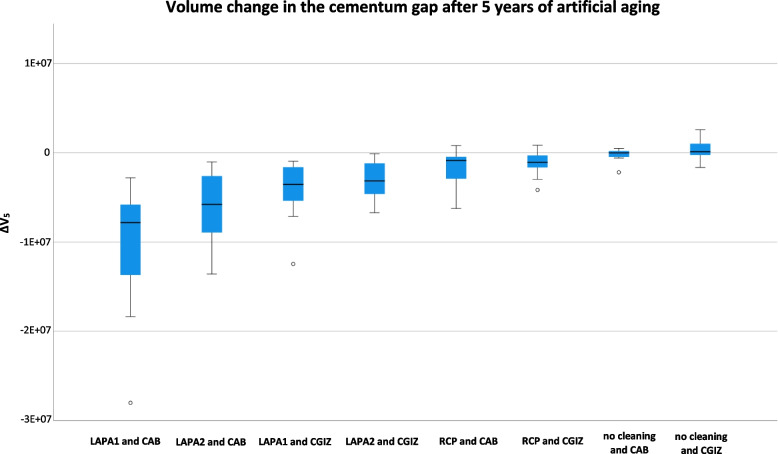
Volume change in the luting gap after 5 years of artificial aging. Boxplot diagram visualizing the volume change (∆V_5_) in the luting gap after 5 years of professional mechanical biofilm removal (PMPR) and artificial aging for all investigated devices (RCP (rubber cup and polishing paste), LAPA-1 (air-polishing device with glycine powder) and LAPA-2 (air-polishing device with erythritol powder) and luting cements (GIZ (glass-ionomer cement) and AD (adhesive bonding) compared to the control group (no cleaning)

Intergroup comparison identified LAPA-1 as having the highest significant abrasion determined on CAB (∆V: -1.05 × 10^7^ (7.2 × 10^6^) µm^3^), followed by LAPA-2 on CAB (∆V: -6.29 × 10^6^ (4.24 × 10^6^) µm^3^), LAPA-1 on CGIZ (∆V: -4.15 × 10^6^ (3.25 × 10^6^) µm^3^), LAPA-2 on CGIZ (∆V: -3.0 × 10^6^ (2.23 × 10^6^) µm^3^), RCP on CAB (∆V: -1.86 × 10^6^ (2.23 × 10^6^) µm^3^) and CGIZ (∆V: -1.2 × 10^6^ (1.31 × 10^6^) µm^3^; (*p* ≤ 0.001)) (Fig. 5).

## Discussion

**Fig. 6:**
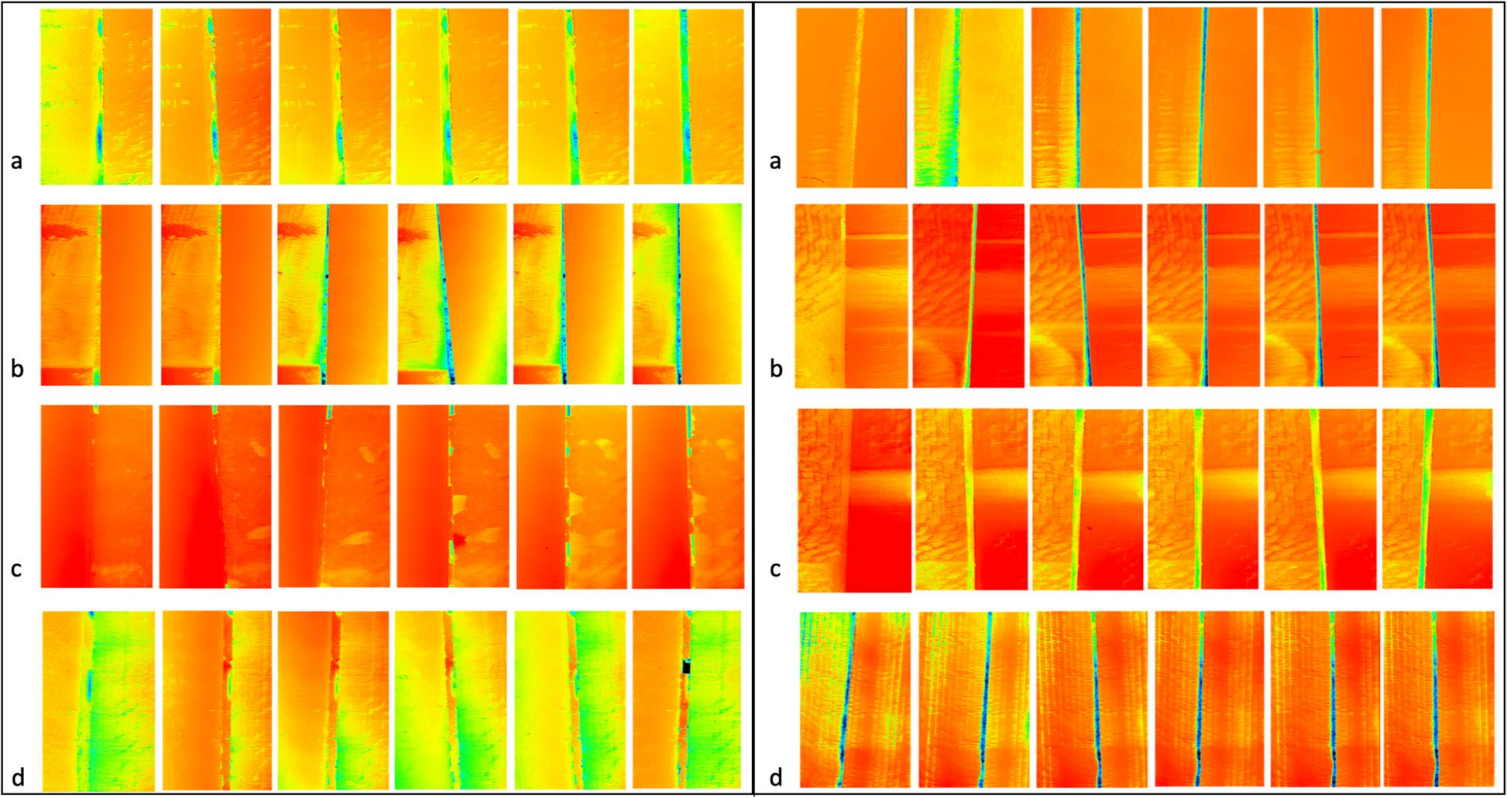
Exemplary illustration of the luting gap over 5 years of artificial aging. Exemplary illustration of the luting gap over 5 years of professional mechanical biofilm removal (PMPR) and artificial aging, on the left side for one crown replica with AD (adhesive bonding) and on the right side with GIZ (glass-ionomer cement) for all investigated devices **a** LAPA-1 (air-polishing device with glycine powder), **b **LAPA-2 (air-polishing device with erythritol powder), **c** RCP (rubber cup and polishing paste) and the **d** control group (no cleaning)

The main objective of this experimental study was to determine the loss of material in a luting gap of ceramic crowns after five years of simulating PMPR using different methods. We found a statistically significant loss of substance in the luting gap compared with the control sides (*p* ≤ 0.001, Table 1). Despite the deficiencies of in vitro studies such as the current work, we found that the highest loss was determined in luting gaps with luting resin bonding by LAPA-2, followed by LAPA-1 and the lowest values were obtained after polishing with rotating rubber cups (Table 1). A possible explanation for the determined effect of volume loss in our study is certainly that due to the long investigation period of more than 5 years of simulated artificial aging as well as the PMPR performed ten times within this period, a summation of the volume losses occurred (see Fig. 6). The null hypothesis of this experimental study must be rejected on the basis of the results obtained, presented and summarized.

This is contrary to other investigations, that found no significant damage to the gingiva or exposed root surfaces regardless of the low abrasive powder used (glycine or erythritol) [24, 25] and up to date, it seems that low-abrasiveness powders for air polishing devices combine minimal abrasion with a maximal cleaning effect [24–26]. Beside the potential risk of air emphysema during use [14], no further (severe) negative side effects were published. Overall, air polishing devices combine various advantages, such as protection of the surrounding tissue; reduced treatment time; high patient acceptance, especially in cases of hypersensitivity; additional antibacterial effects (powder dependent); and reduced noise [27]. However, we have not investigated the cleaning efficacy, but we assumed that air polishing versus conventional rotating polishing would not differ from a clinical point of view [25, 28] and would be utilized by dental auxiliaries comparably in daily routine during SPT [14]. Nevertheless, a paradigm shift has positioned AP as the perceived standard for SPT [15]. The two tested air polishing devices utilize erythritol powder (LAPA-1) or glycine (LAPA-2), which differ not only in the average particle size but also in their chemical nature [26] and will influence their cleaning performance [29]. It should be noted that biofilm removal in SPT should not exert any harmful effects on the root surface [8] and both tested LAPA-1 and LAPA-2 were approved for subgingival cleaning. They were designed in such a way that the powder-water jet was directed vertically onto the root surface to reduce what is known as the "flow pressure" [29] and is thought to contribute to particularly gentle supra- and subgingival PMPR. We observed that utilizing AP subgingival or epigingival is easier versus RCP, however, the risk of having side effects due to the specific characteristics of the powder jet has to be assumed [30]. Some specific properties of the cements used in the study result from the requirements placed on these luting materials. In this study, replicas cemented with GIZ (mechanical retention mechanism) and resin cement (AD) (both micro-mechanical and chemical retention mechanisms) were investigated. While the cements should primarily exhibit mechanical stability, they must also be viscous enough to ensure adequate spreading. At the same time, the film thickness must not be so high that the fit of the restoration is compromised.

The lower proportion of fillers in resin cements compared to restorative composites also affects their mechanical properties [31]. The requirement to be sufficiently viscous is, to some extent, opposed to the requirement for mechanical stability. Primarily, cements are not designed to absorb high mechanical energy as it is applied to them in the form of air polishing devices. Furthermore, it can be assumed that fillers are dissolved out of the matrix by the test method but that the spaces created are refilled by the subsequent processing with powders containing even very small particles. The fact that GIZ shows some expansion due to water absorption has already been reported [32]. This provides a possible explanation for the results of the control group (overall volume increase) without surface treatment. The measured increase in the width of the luting gap could be explained by a clearer delineation of the cement and ceramic surfaces after cleaning compared to baseline. Thus, it can be assumed that the deviation in the measurement from baseline to end has an optical cause.

In addition to the details discussed before, our experimental study has further limitations. Due to the nature of any in vitro simulation, the results cannot be transferred directly to clinical situations in general and further studies must be performed in a clinical setting in the future. In our experimental study, the aging of the material was simulated, but without the stress of mastication. This would place additional mechanical stress on the cement. After brushing the luting gap with a sonic brush for five seconds, no further mechanical treatment of the surfaces was considered within the context of a home oral hygiene regimen. Another limitation is that the working angle of the powder jet ranged from 0—90°, simulating a worst-case scenario with maximum exposure of the powder to the luting gap. In addition, although there was extensive training and clinical calibration with different methods of PMPR between the two practitioners, no interrater reliability could be determined due to the methods used. A last critical point is that the replicas were removed from the water bath for cleaning and profilometric measurement, during which the specimens (partially) dried out. These multiple alternations of drying and re-wetting may represent further mechanical stress on the material. Nevertheless, the presented in vitro analyses enabled the reproducible investigation of defined parameters that cannot be measured clinically, thus increasing the sensitivity of the comparisons.

## Conclusions

Within the limitations of the present experimental study, it can be concluded that all PMPRs caused a significantly higher substance loss in the luting gap, independent of the used luting material. The highest loss was determined at resin cements by air- versus rotating rubber cup polishing after simulated five years of SPT, awareness should be raised for the risk of decementation or secondary caries in long-term. However, these have to be evaluated by future clinical studies.

## Data Availability

The datasets used and analyzed during the current study are not publicly available due to [national data protection law] but are available from the corresponding author on reasonable request.

## Abbreviations

AD: Adhesive bonding
AP: Air polishing
GIZ: Glass-ionomer cement
CAB: Crown replicas with adhesive bonding
CGIZ: Crown replicas with glass-ionomer cement
LAPA: Low-abrasiveness powder air polishing
PMPR: Professional biofilm removal
RCP: Rubber cup polishing
SD: Standard deviation
SPT: Supportive periodontal therapy
